# The Clinical Validation of the Athlete Sleep Screening Questionnaire: an Instrument to Identify Athletes that Need Further Sleep Assessment

**DOI:** 10.1186/s40798-018-0140-5

**Published:** 2018-06-04

**Authors:** Amy M. Bender, Doug Lawson, Penny Werthner, Charles H. Samuels

**Affiliations:** 1 Centre for Sleep & Human Performance, 106-51 Sunpark Dr. SE, Calgary, AB T2X 3V4 Canada; 2 Faculty of Kinesiology, University of Calgary, Calgary, AB Canada; 3 Faculty of Chiropractic, D’Youville College, Buffalo, NY USA

**Keywords:** Elite athletes, Sleep disturbances, Sleep interventions

## Abstract

**Background:**

Previous research has established that general sleep screening questionnaires are not valid and reliable in an athlete population. The Athlete Sleep Screening Questionnaire (ASSQ) was developed to address this need. While the initial validation of the ASSQ has been established, the clinical validity of the ASSQ has yet to be determined. The main objective of the current study was to evaluate the clinical validity of the ASSQ.

**Methods:**

Canadian National Team athletes (*N* = 199; mean age 24.0 ± 4.2 years, 62% females; from 23 sports) completed the ASSQ. A subset of athletes (*N* = 46) were randomized to the clinical validation sub-study which required subjects to complete an ASSQ at times 2 and 3 and to have a clinical sleep interview by a sleep medicine physician (SMP) who rated each subjects’ category of clinical sleep problem and provided recommendations to improve sleep. To assess clinical validity, the SMP category of clinical sleep problem was compared to the ASSQ.

**Results:**

The internal consistency (Cronbach’s alpha = 0.74) and test-retest reliability (*r* = 0.86) of the ASSQ were acceptable. The ASSQ demonstrated good agreement with the SMP (Cohen’s kappa = 0.84) which yielded a diagnostic sensitivity of 81%, specificity of 93%, positive predictive value of 87%, and negative predictive value of 90%. There were 25.1% of athletes identified to have clinically relevant sleep disturbances that required further clinical sleep assessment. Sleep improved from time 1 at baseline to after the recommendations at time 3.

**Conclusions:**

Sleep screening athletes with the ASSQ provides a method of accurately determining which athletes would benefit from preventative measures and which athletes suffer from clinically significant sleep problems. The process of sleep screening athletes and providing recommendations improves sleep and offers a clinical intervention output that is simple and efficient for teams and athletes to implement.

**Electronic supplementary material:**

The online version of this article (10.1186/s40798-018-0140-5) contains supplementary material, which is available to authorized users.

## Key Points

- When athletes were rated based on the category of clinical sleep problem, there was good agreement between the ASSQ scoring system and the sleep medicine physician.
- Twenty-five percent of athletes were identified as needing further clinical sleep assessment. This is much lower than general sleep screening questionnaires which have not been validated in elite athletes.
- Sleep improved from baseline to after the sleep recommendations in those athletes with moderate to severe sleep difficulty. The ASSQ provides a valid and reliable tool to identify athletes for further sleep assessment and is easy and efficient for athletes and the support team to administer.

## Background

Sleep is a fundamental biological process that facilitates recovery from the mental and physical demands of high-performance sport [1, 2]. Recently, there has been a proliferation of research exploring how sleep impacts recovery, training, and performance in elite athletes. Previous research has indicated elite athletes have a high prevalence of poor sleep quality [1, 3–8] and insufficient sleep quantity [9–11]. However, the quality of the research has been hampered by the investigative methods [3].

In particular, the Pittsburgh Sleep Quality Index (PSQI), which is the primary questionnaire used to assess sleep in athletes [12], has not been validated in an athlete population [13, 14], is difficult to score, lacks information specific to athletes, and shows poor concordance rates with the clinical assessment of a sleep medicine physician (SMP; [13]). Another tool, the Athlete Sleep Behavior Questionnaire (ASBQ), is used to identify maladaptive sleep behaviors in athletes [15]. The ASBQ shows promise to differentiate sleep behaviors between athletes and controls but is still in development to determine valid cut-points that are not based on the authors’ speculation. Furthermore, the ASBQ is not intended to be used as a clinical sleep screening tool but instead to inform sleep hygiene recommendations for athletes.

The sport science community considers sleep to be an important part of the recovery process [16]; therefore, it is important to have a valid and reliable questionnaire that can be used as a first-line tool to screen and identify athletes with clinically relevant sleep problems and possible sleep disorders. This allows quick intervention only when necessary and differentiates those who may only require education and behavioral interventions.

The Athlete Sleep Screening Questionnaire (ASSQ) was developed as a sleep screening tool to detect clinically significant sleep disturbances and daytime dysfunction and to provide interventions based on the type and severity of the problem that is detected in an athlete population [13]. The details of the initial development of the ASSQ have been previously published [13]. Briefly, a 15-item questionnaire was developed to assess both sleep and circadian factors of sleep quantity, sleep quality, insomnia, and chronotype with a timeframe of “over the recent past.” Answers from the first seven questions were compiled to create a sleep difficulty score (SDS) which categorized athletes into four categories of clinical sleep problems—none, mild, moderate, and severe. The SDS system did not take into consideration chronotype (four questions) or other important factors of sleep-disordered breathing and sleep and performance during travel, but were used to guide the SMP as to who required follow-up and further diagnostic testing. Based on the previous SDS cutoffs, 13% of the 349 athletes studied were classified as having moderate to severe clinical sleep problems and required intervention recommendations from a SMP [13]. However, no formal analyses of the clinical validity of the ASSQ were performed.

To address the lack of clinical validation, 199 Canadian National Team athletes completed the ASSQ with 46 athletes randomized to partake in a standardized clinical sleep interview from a SMP who was blinded to the ASSQ responses and SDS. At the end of the interview, the SMP classified athletes into the level of clinical sleep problem, and those classifications were compared to the new ASSQ scoring system to determine the clinical validity of the questionnaire. This manuscript describes the current version of the ASSQ and the methodology used to determine the reliability of the instrument and the clinical validity. In addition, the process of making sleep recommendations based on the category of clinical sleep problem is described.

## Methods

### Participants and Procedures

Canadian National Team carded athletes (*N* = 199) from both senior national teams and lower-level national teams participated in the study. The athletes were between the ages of 18–36 from 23 different summer and winter sports. The core questionnaire was the same as previously published [13]; however, an additional question on caffeine consumption was added, and a subset of athletes had one additional question on electronic device use, see Additional file 1. The consent form was located on the first page of the online survey (www.surveymonkey.com), and if participants continued to complete the survey, it was an indication of their consent to participate. The study was performed in accordance with the standards of ethics outlined in the Declaration of Helsinki and approved by the University of Calgary Conjoint Health Research Ethics Board.

### Clinical Validation Sub-study

**Fig. 1:**
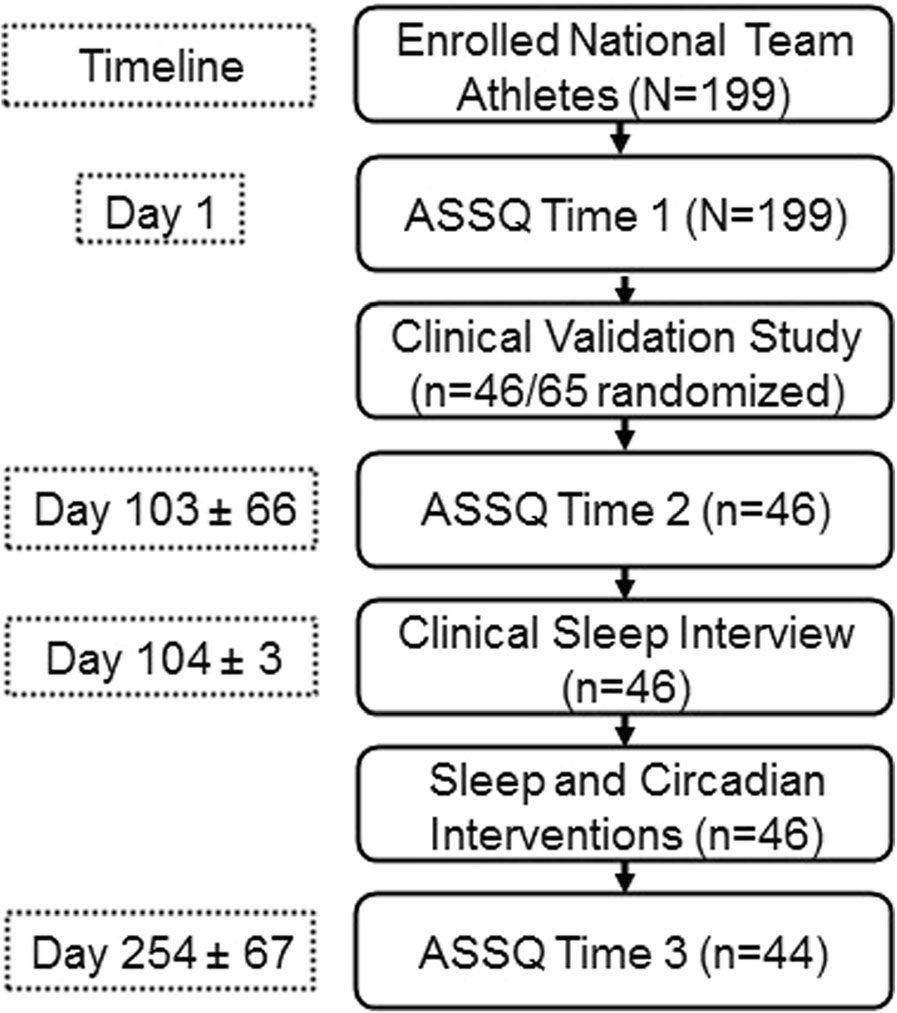
Study timeline and protocol. Cumulative days (mean ± SD) elapsed in the study (left dot boxes) between protocol procedures (right solid boxes)

Following the completion of the initial ASSQ at time 1 (T1), 65 athletes were randomized to the clinical validation sub-study. Seventy-one percent of the athletes (*N* = 46) could comply with the requirements of the protocol. The protocol included 2 weeks of wrist-watch actigraphy, which is not presented here (Readiband, Fatigue Science, Canada; [17]), an ASSQ completed at time 2 (T2), a standardized clinical sleep interview with the SMP, a rating of the category of clinical sleep problem from the SMP, a more detailed follow-up interview if required, recommendations for sleep interventions, and an ASSQ completed at time 3 (T3), see Fig. 1.

Once an athlete was randomized to the clinical validation sub-study, they were contacted to determine the actigraphy recording period which started after at least 1 day for every time zone traveled recently to accommodate for any circadian misalignment from recent travel. After the recording period, athletes completed the ASSQ T2 to assess test-retest reliability which occurred (102 ± 66 days) after T1 but prior (1 ± 3 days) to the structured clinical sleep interview with the SMP. The structured clinical sleep interview was performed online through videoconferencing or over the phone. The SMP followed a standardized interview sheet which was based on prior clinical experience working with athletes. It included questions about sleep history (e.g., do they think they have a sleep problem), estimated sleep parameters (e.g., sleep duration, naps, sleep latency, wake after sleep onset), sleep disorders (e.g., insomnia, delayed sleep phase syndrome, sleep-disordered breathing, periodic limb movement, parasomnias, and bruxism), timing of sleep (e.g., preferred bedtime and wake time, do they see themselves as a morning-type or evening-type), and if the athlete had issues with sleep or performance during travel. After the interview was completed (average duration 9.9 ± 3 min), the SMP who was blinded to the results from the ASSQ at T1 and T2 rated the category of clinical sleep problem based on how severe the athletes sleep disturbances were (none, mild, mild to moderate, moderate, moderate to severe, and severe) and indicated which of the standardized recommendations were to be communicated to the subject.

### Sleep Intervention Recommendations

Athletes were emailed results based on the recommendations from the SMP after the questionnaires were examined (those athletes not randomized *N* = 153) or after the interviews (*N* = 46). In those who the SMP classified as moderate to severe (*N* = 16), a second more detailed follow-up interview to evaluate the problem further and to discuss the recommendations occurred. The recommendations were standardized based on the SMP’s rating of clinical sleep problem and the responses to the modifier questions. The recommendations included a general sleep education sheet on sleep quantity, quality, timing, and proper sleep hygiene for all the athletes. Also included were individualized recommendations depending on their responses to sleep duration, napping activity, insomnia, and sleep-disordered breathing symptoms. If indicated, a travel and jet lag fatigue management education sheet, recommendations for an insomnia self-help book or standardized online CBT-Insomnia program, a circadian re-entrainment program using light therapy and melatonin, and recommendations for further sleep testing or treatment in their local area were provided. When possible and with the athlete’s consent, the sport physician and lead integrated support team members would also get the results to help monitor the athlete’s progress on a more frequent basis. All but two athletes in the clinical validation sub-study completed an ASSQ T3 approximately 150 ± 67 days after the clinical interview to assess if the sleep recommendations helped improve sleep as reflected in a reduction in the SDS.

### Statistical Analyses

To assess the sample characteristics, descriptive statistics were determined for age, sex, sport, status on the senior national team or lower-level national team, years at the current level, and training season the questionnaire was completed in. The median time taken to complete the ASSQ (generated from www.surveymonkey.com) was also assessed.

The internal consistency of the ASSQ items was estimated using Cronbach’s alpha. Test-retest correlations compared the stability of the scale from the T1 to T2, prior to the recommendations taking place.

To determine if the questions for each scale could be summed for a score, unidimensionality of the latent trait being measured (sleep difficulty and chronotype) was assessed using principal component analysis (PCA) [18]. Exploratory factor analysis (EFA) was performed on both the sleep difficulty score and chronotype score. Pearson’s correlation coefficients were estimated between the original sleep difficulty score and the new sleep difficulty score. Confirmatory factor analysis (CFA) was performed on both sleep difficulty and chronotype scores.

To determine if the sleep difficulty score and chronotype score were consistent across different groups, the data from the Canadian National Team athletes was compared to the data from a separate study in *N* = 1074 competitors from the London Virgin Money Marathon who completed an expanded version of the ASSQ (manuscript in preparation). For data from Canada and the UK, the CFA path coefficients were compared, as well as the item correlations.

Comparisons of level of clinical sleep problem between the ASSQ and the SMP were performed using weighted kappa [19–21]. Weighted kappa was used as kappa (unweighted) does not consider the degree of disagreement. Reliability was estimated for the ASSQ scoring system with Cronbach’s alpha. Sensitivity, specificity, positive predictive value, and negative predictive value [22] were estimated from the ASSQ scoring system sleep problem categories to the SMP categories after the clinical interview. Sensitivity was estimated as the number of subjects determined by the ASSQ scoring system as needing a clinical intervention from the SMP (moderate and severe) divided by the number of subjects needing a clinical intervention as determined by the SMP. Specificity was estimated by the number of subjects determined by the ASSQ scoring system as not needing a clinical sleep intervention (none, mild categories) divided by the number of subjects not needing a clinical intervention as determined by the SMP. The positive predictive value was estimated by dividing the subjects for which there was agreement on needing a clinical intervention (moderate, severe categories) by the actual number of subjects needing a clinical intervention as determined by the SMP. The negative predictive value was estimated by dividing the subjects for which there was agreement on not needing a clinical intervention by the actual number of subjects not needing a clinical intervention as determined by the SMP.

To assess the impact of the sleep recommendations, simple paired *t* tests were performed at T1 (baseline) and T3 (post-recommendations) for the SDS and the categories of clinical sleep problem.

All statistical analyses were performed with R 3.3.3 (R Core Team, Vienna, Austria). Descriptive statistics, Cronbach’s alpha exploratory factor analysis and correlation estimates were performed with the Psych package [23]. CFA were performed with the Lavaan package [24]. Inter-rater estimates and weighted kappa were performed with the IRR package [25].

## Results

### Sample Characteristics

The ASSQ was administered to 199 Canadian National Team carded athletes. The athletes were between the ages of 18–36 (mean age 24.0 ± 4.2 years) with 62% (*N* = 123) of the sample females. The sample included representation of athletes from 23 different summer and winter sports, see Table 1. Eighty-one percent (*N* = 162) of the sample was on the senior national team of their sport with the remainder (*N* = 37) carded but on lower-level national teams. Sixty-seven percent of the sample (*N* = 133) had 5 years or less experience at the national team level, and 9% (*N* = 19) had been at their current level for 10 years or more. The majority of athletes (68%) completed the ASSQ during their competitive season with 24% in pre-season and 8% during their rest season.

**Table 1:** Participant characteristics

Sport	*N*	Sex F	Age	Years at level
		*N* (%)	Mean ± SD	Mean ± SD
Alpine skiing	16	6 (38)	20.6 ± 3.0	2.8 ± 2.3
Athletics^a^	26	19 (74)	26.3 ± 4.1	4.5 ± 3.2
Basketball	9	9 (100)	25.4 ± 5.1	6.3 ± 4.9
Biathlon	10	5 (50)	25.4 ± 2.9	5.1 ± 3.1
Canoe/kayak	1	0 (0)	31	11
Cross-country skiing	10	4 (40)	25.7 ± 4.9	5.3 ± 3.9
Cycling	6	5 (83)	27.3 ± 4.5	6 ± 4.8
Diving	4	1 (25)	23.3 ± 3.1	5.0 ± 1.4
Field hockey	22	22 (100)	22.5 ± 2.8	3.8 ± 2.3
Figure skating	12	6 (50)	24.5 ± 4.3	5.7 ± 3.1
Freestyle skiing^a^	45	22 (49)	22.8 ± 3.6	4.6 ± 2.8
Golf	14	5 (36)	21.6 ± 2.5	3.2 ± 2.2
LT speed skating	1	0 (0)	24	4
Luge^a^	5	4 (80)	27.4 ± 6.8	6.8 ± 5.2
Soccer^a^	6	6 (100)	27.7 ± 4.9	6.8 ± 5.7
Swimming	3	2 (66)	20.1 ± 3.7	3.7 ± 3.8
Triathlon	5	4 (80)	21.2 ± 3.3	2.3 ± 1.2
Wrestling	2	2 (100)	29.5 ± 6.4	7.5 ± 3.5

*F* female, *LT* long track

^a^One athlete from modern pentathlon included in athletics, one athlete from skeleton included in luge, one athlete from rugby included in soccer, two athletes from ski jumping and two athletes from snowboarding included in freestyle skiing

The median time to complete the survey was 5 min (range 1 to 268 min). There were four athletes who took longer than 60 min to complete the survey (73, 186, 191, and 268 min). It was assumed this was not a true measure of the time to complete the survey continuously; therefore, the median was used as a more appropriate measure of the time taken rather than the average time completed.

### Internal Consistency

The internal consistency of the seven ASSQ items that made up the SDS was poor at T1 (Cronbach’s alpha = 0.58; 95% CI 0.50 to 0.66). The two napping questions related to how often the athlete napped and the duration of the nap correlated poorly with the sleep difficulty score *r* = 0.16 and *r* = 0.04, respectively. Reliability and exploratory factor analysis were then repeated without these questions. With the nap questions removed, the internal consistency of the five items was acceptable (Cronbach’s alpha = 0.74; 95% CI 0.69 to 0.79). The average correlation with the total score was *r* = 0.69 for the five items with the lowest correlation for medication use (*r* = 0.42; item 6) and the highest correlation for satisfaction with quality of sleep (*r* = 0.85, item 3). The factor loadings based on the EFA (one factor, varimax rotation) for the SDS five items were item 1 = 0.56, item 3 = 0.87, item 4 = 0.57, item 5 = 0.68, and item 6 = 0.27, with 40% of the variance explained. Although item 6 loaded weakly on the sleep difficulty factor, it was not dropped from the scale as it positively contributed to the reliability measure. The PCA of the new SDS revealed a strong first component (2.48) with all other components having eigenvalues of less than 1.0, which indicated the scale is unidimensional and can be summed for a score. The correlation between the new 5-item SDS with the 7-item SDS was strong (*r* = 0.97, 95% CI 0.96 to 0.98). The duration of the nap question was dropped from the questionnaire, but the nap frequency question (see Additional file 1, item 2) was kept in the questionnaire to inform sleep education strategies.

The internal consistency of the four questions from the chronotype score (items 7–10) was acceptable (Cronbach’s alpha = 0.73; 95% CI 0.67 to 0.79). The average correlation with the total score was *r* = 0.77 for the four items with the lowest correlation for preferred time to bed (*r* = 0.64; item 10) and the highest correlation for self-reported chronotype (*r* = 0.86, item 9). The factor loadings for the chronotype four items based on the EFA (one factor, varimax rotation) were item 7 = 0.66, item 8 = 0.52, item 9 = 0.88, and item 10 = 0.51, with 44% of the variance explained. The PCA of the chronotype items revealed a strong first component (2.23) with all other components having eigenvalues of less than 1.0 which indicated the scale is unidimensional and can be summed for a score.

The time between completing ASSQ T1 to T2 was 101 ± 66.2 days. There was a strong relationship (*r* = 0.86, 95% CI 0.75–0.92) for the 5-item SDS from T1 to T2, indicating good stability of the SDS. There was a strong relationship (*r* = 0.78, 95% CI 0.63–0.87) for the 4-item chronotype factor from T1 to T2 indicating good stability of the chronotype score.

### Stability of the ASSQ Across Populations

CFA revealed comparable factor loadings on the SDS items for the Canadian National Team athletes to the London Marathon runners respectively (0.56 vs 0.42, item 1; 0.88 vs 0.82, item 3; 0.57 vs 0.39, item 4; 0.68 vs 0.63, item 5; 0.27 vs 0.25, item 6). The comparative fit index (CFI) was 0.98, and the root mean square error of approximation (RMSEA) was 0.06 (95% CI 0.00 to 0.13). There was also good stability across the populations for the chronotype score. CFA revealed comparable factor loadings for both populations (0.65 vs 0.52, item 7; 0.52 vs 0.52, item 8; 0.88 vs 0.90, item 9; 0.51 vs 0.43, item 10), with a CFI of 0.95 and a RMSEA of 0.15 (95% CI 0.07 to 0.24). For the Canadian data, the average correlation was 0.35. The smallest correlation was between item 1 (sleep quantity) and item 6 (use of sleep medication). The largest correlation was between item 3—being satisfied with sleep quality—and item 5—trouble staying asleep. For the London Marathon data, the average correlation was 0.25. The smallest and largest correlations were the same relationships as those seen in the Canadian National Team data.

### Clinical Validity

Cut-points were made to the new 5-item SDS to categorize athletes into clinical sleep problem of none (0–4), mild (5–7), moderate (8–10), and severe (11–17). When the SMP’s ratings of level of the clinical problem were compared to the ASSQ scoring system (see Additional file 2), the groups were not shown to be different (chi-square = 0.23, df = 3, *p* = 0.97), see Table 2. Agreement between the two rating systems was good (Cohen’s weighted kappa = 0.84, *z* = 5.68, *p* < 0.01). The sensitivity of the ASSQ to detect clinically meaningful sleep problems (moderate to severe category) was 81% (95% CI 54.4 to 96.0%) when compared to the SMP ratings. The specificity of the ASSQ scoring system to categorize athletes as not needing follow-up with the SMP (none to mild clinical sleep problem) was 93% (95% CI 77.9 to 99.2%). The positive predictive value of the ASSQ scoring system was 92% (95% CI 63.1 to 98.8%). The negative predictive value of the ASSQ scoring system was 90% (95% CI 77.1 to 96.3%). When the ASSQ scoring system was applied to the entire sample, 25.1% of athletes were identified as having a moderate or severe level of clinical sleep problem and recommendations were made for further follow-up.

**Table 2:** ASSQ scoring system vs. sleep medicine physician ratings

ASSQ scoring system					
SMP rating	None	Mild	Moderate	Severe	Total
None	15	4	0	0	19
Mild	3	6	2	0	11
Moderate	0	3	9	1	13
Severe	0	0	1	2	3
Total	18	13	12	3	

Categories of clinical sleep problem between the sleep medicine physician (rows) and the ASSQ (columns). Perfect agreement is along the diagonal

### Impact on SDS After Sleep Recommendations

**Fig. 2:**
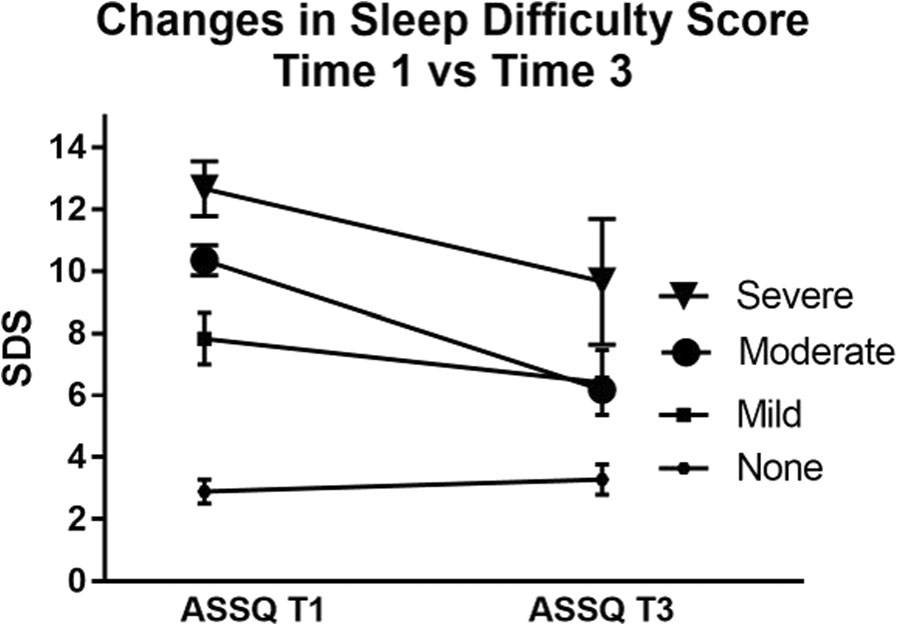
Changes in sleep difficulty score for each clinical sleep problem category (none, *n* = 18; mild, *n* = 12; moderate, *n* = 11, and severe, *n* = 3) from time 1 (T1) at baseline to time 3 (T3) after the sleep intervention recommendations

For all athletes in the sub-study who completed T3 (*N* = 44), there was an average reduction of SDS from T1 to T3 of 1.5 points (*t* = 1.93, df = 80.72, *p* = 0.06). When the changes for each of the groups were assessed (see Fig. 2), the athletes who were in the none category did not improve after the recommendations (*t* = 0.78, df = 17, *p* = 0.45). This is likely because there was no clinical sleep problem present. Those classified as having a mild clinical sleep problem had an average drop of 1.4 points on the SDS, but this was not statistically significant (*t* = 0.78, df = 17, *p* = 0.11). Athletes who had a moderate (*N* = 11) or severe (*N* = 3) level of clinical sleep problem showed the greatest improvement after the recommendations with an average drop of 3.9 points on the SDS (*t* = 5.75, df = 13, *p* < 0.01), see Fig. 2.

## Discussion

The primary objective of this study was to assess the clinical validity of the ASSQ. After a standardized clinical sleep interview, the SMP categorized athletes into levels of sleep problem and this was compared to the ASSQ rating system. There was no significant difference between the ratings of the ASSQ and the SMP, which showed good agreement between the categories, see Table 2. The ASSQ had good specificity of 93% but only an acceptable sensitivity of 81%. The measure of sensitivity did not take into consideration the modifiers which is the second part of the ASSQ’s scoring system, see Additional file 2. In 10 out of 11 cases where the SMP rated the athlete as having a higher category of sleep problem than the ASSQ, there were modifiers present. The reason the sleep-disordered breathing and evening-type modifiers were not included in the SDS was because the psychometric properties of the questionnaire would be negatively impacted because of a low prevalence of these problems occurring in athletes [26, 27]. However, case finding is critical because of the clinical significance, so having these as a part of the secondary scoring system is important. For the questions related to travel, this was not included in the SDS because not all athletes travel. It was included as a modifier to identify athletes who could benefit from specific interventions to help sleep disturbance during travel. Therefore, the SDS in conjunction with the modifiers should be used to determine the degree of the sleep problem and guide the intervention strategy; see Additional file 2. The other three items on napping frequency (item 2), caffeine use (item 15), and electronic device use (item 16) were not part of the scoring system but were kept in the ASSQ to inform sleep optimization strategies.

Another objective of the current study was to assess the psychometric properties of the ASSQ. After the two nap questions were removed from the SDS, the internal consistency of the new 5-item SDS was acceptable. Test-retest reliability from T1 to T2 over a period of 101 days showed good stability (*r* = 0.86). The ASSQ also was stable across two different populations when the Canadian National Team data were compared to a larger set of runners (*N* = 1074) from the 2016 London Virgin Money Marathon.

In the current study, we found that 25% of athletes in the sample were identified as having clinically relevant sleep problems. This is similar to Tuomilehto et al. who found approximately 21% of a sample of 107 professional Finnish hockey players have a sleep disorder as verified by polysomnography [6]. However, when compared to studies which utilized the PSQI [12], our prevalence of athletes with sleep problems was much lower than previous studies which showed 40–50% of athletes had poor sleep [1, 4, 5, 7, 8]. A recent study confirmed poor concordance rate of the PSQI with the sleep evaluation of athletes by a SMP [13]. The discrepancies between the ASSQ and the PSQI could be due to the PSQI not being validated in an athlete population. Athletes are exposed to extensive monitoring of symptoms and could be more sensitive to reporting symptoms than the general population [14]. This has important implications for both the research methodology and the clinical use of the PSQI in an athlete population. Over-identifying athletes that need clinical sleep interventions is inefficient and expensive, and those athletes with more severe sleep issues may not get interventions in a timely manner. The ASSQ can be easily deployed by the athletes’ support staff and can quickly identify those who need further assessment and treatment. By utilizing the proper sleep intervention recommendations, athletes can begin to reduce sleep disturbances and optimize sleep. Additionally, the sport and sleep science communities now have a valid and reliable sleep screening tool to use in this unique population.

### Limitations

The most significant methodological limitation of this study was the choice to use one SMP as the “gold standard” by which the clinical validity of the ASSQ was evaluated. Ideally, the study would have used multiple SMPs to rate each athlete and subsequently perform inter-rater reliability testing. This research did not include another SMP because of the limited number of SMPs who specialize in evaluating sleep disturbances and sleep-related dysfunction in elite athletes. The decision to begin the exploration of the clinical validity of the ASSQ using one SMP allowed the researchers to start the process, and we certainly encourage further validity and reliability testing of the ASSQ.

Although the chronotype score showed good psychometric properties, one limitation in this study was that it was not validated with existing chronotype questionnaires or biological markers of circadian phase. Future research could test the ASSQ chronotype score against existing questionnaires for cut-points of morningness and eveningness and verify these cut-points against biological markers of circadian phase.

Another limitation of our study was the lack of objective markers of sleep disturbance. The protocol did include actigraphy over a 2-week period; however, the purpose of the study was to examine the clinical validity of the questionnaire, which actigraphy cannot assess. Polysomnography may have been more appropriate to confirm the presence of sleep disorders but is typically only one night of data whereas the questionnaire asks about sleep patterns across a much longer period (“the recent past”). In addition, the feasibility of using polysomnography in this study would have been extremely difficult because athletes participated from places across Canada, and a minority of the athletes were training and competing in locations around the world. Again, the purpose of the questionnaire is to identify athletes needing further sleep assessment and is not intended to diagnose athletes with sleep disorders.

### Instructions for ASSQ Usage (See Additional file 2)

It is recommended to assess the SDS first (items 1, 3, 4, 5, and 6), then the modifiers of sleep-disordered breathing (items 13 and 14), travel (items 11 and 12), and chronotype (items 7–10), and finally evaluate the items of interest (e.g., item 2, 15, 16) to inform more specific sleep optimization strategies.

#### Sleep Difficulty Score (SDS)

The SDS is used to classify athletes into the level of clinical sleep problem (none, mild, moderate, severe) based on the response to items 1, 3, 4, 5, and 6 with poorer sleep indicative of a higher score. Responses to those items are summed, and scores of 0–4 are classified as the “none” category, scores of 5–7 are classified as the “mild” category, scores of 8–10 are classified as the moderate category, and scores of 11–17 are classified as the severe category. Those athletes classified as having a moderate or severe sleep problem should be further evaluated.

#### Modifiers

The modifiers are not included in the SDS because they occur less frequently and are not always applicable to certain athlete groups (e.g., travel). They are important to provide specific education and intervention recommendations.

##### Sleep-Disordered Breathing

If an athlete answers yes to item 13 (loud snoring) or item 14 (sleep apnea), they should be further evaluated.

##### Travel

If the athlete answers yes to item 11 (sleep disturbance), education on travel management is recommended. If the athlete answers yes to item 12 (performance issues), the problem is likely more serious and may require further assessment and treatment.

##### Chronotype

Sleep difficulty is more common in athletes who are evening types. Add the scores from items 7–10 to get the chronotype score for eveningness. Totals ≤ 4 indicate the athlete is an evening type and may require further assessment and treatment (e.g., bright light therapy, melatonin).

#### Items of Interest

Items of interest use the responses to specific items to inform sleep optimization strategies. For example, if an athlete is only getting 6–7 h of sleep (item 1), and not napping frequently throughout the week (item 2), a recommendation to increase nighttime sleep duration and napping would be warranted.

## Conclusions

The psychometric properties of the data (reliability, test-retest, and association with independent expert judgment) suggest strongly that the ASSQ can detect clinically meaningful sleep disturbances in an elite athlete population. The ASSQ is easy to administer, quick to complete, and can be scored remotely. Most importantly, it provides support staff the capability to understand when further follow-up with a SMP or qualified sleep professional is necessary. We found that the prevalence of clinically meaningful sleep disturbances was much lower than with previously used tools and caution researchers and clinicians about using tools that have not been properly validated in an athlete population. With sleep screening, recommendations, and proper follow-up, athletes can improve their sleep for the benefit of better health and performance.

## Additional File

Additional file 1: Athlete Sleep Screening Questionnaire (ASSQ). (PDF 13 kb)

Additional file 2: ASSQ sleep difficulty score (SDS) scoring key. (DOCX 22 kb)

## Abbreviations

ASSQ: Athlete Sleep Screening Questionnaire
CFA: Confirmatory factor analysis
CFI: Comparative fit index
EFA: Exploratory factor analysis
PCA: Principal component analysis
RMSEA: Root mean square error of approximation
SDS: Sleep difficulty score
SMP: Sleep medicine physician
T1: Time 1
T2: Time 2
T3: Time 3
