# Supplementary material for: The Clinical Validation of the Athlete Sleep Screening Questionnaire: an Instrument to Identify Athletes that Need Further Sleep Assessment
Source: Sports Med Open. 2018 Jun 4;4:23. doi: 10.1186/s40798-018-0140-5 (PMC5986689; doi:10.1186/s40798-018-0140-5)
Supplement: Supplementary file 1 — Athlete Sleep Screening Questionnaire (ASSQ). (PDF 13 kb) [file 40798_2018_140_MOESM1_ESM.pdf]

Appendix A.

Athlete Sleep Screening Questionnaire (ASSQ)

INSTRUCTIONS

The following questions relate to your sleep habits. Please circle the best answer which you think represents your typical sleep habits over the recent past. For all questions, circle a letter from 'a' to 'e' unless otherwise specified.

1. During the recent past, how many hours of actual sleep did you get at night? (This may be different than the number of hours you spent in bed.)
   a. 5 to 6 hours
   b. 6 to 7 hours
   c. 7 to 8 hours
   d. 8 to 9 hours
   e. more than 9 hours

2. How many naps per week do you take?
   a. none
   b. once or twice
   c. three or four times
   d. five to seven times

3. How satisfied/dissatisfied are you with the quality of your sleep?
   a. very satisfied
   b. somewhat satisfied
   c. neither satisfied nor dissatisfied
   d. somewhat dissatisfied
   e. very dissatisfied

4. During the recent past, how long has it usually taken you to fall asleep each night?
   a. 15 minutes or less
   b. 16 – 30 minutes
   c. 31 – 60 minutes
   d. longer than 60 minutes

5. How often do you have trouble staying asleep?
   a. none
   b. once or twice per week
   c. three or four times per week
   d. five to seven days per week

6. During the recent past, how often have you taken medicine to help you sleep (prescribed or over-the-counter)?
   a. none
   b. once or twice per week
   c. three or four times per week
   d. five to seven times per week

7. Considering only your own “feeling best” rhythm, at what time would you get up if you were entirely free to plan your day?
   a. 5:00 am – 6:30 am
   b. 6:30 am – 7:45 am
   c. 7:45 am – 9:45 am
   d. 9:45 am – 11:00 am
   e. 11:00 am – 12:00 pm (noon)

8. How alert do you feel during the first half-hour after having awakened?
   a. not at all alert
   b. slightly alert
   c. fairly alert
   d. very alert

9. Do you consider yourself to be a morning type person or an evening type person?
   a. definitely a morning type
   b. more a morning type than an evening type
   c. more an evening type than a morning type
   d. definitely an evening type

10. Considering your own “feeling best” rhythm, at what time would you go to bed if you were entirely free to plan your evening?
   a. 8:00 pm – 9:00 pm
   b. 9:00 pm – 10:15 pm
   c. 10:15 pm – 12:30 am
   d. 12:30 am – 1:45 am
   e. 1:45 am – 3:00 am

11. When you are travelling for your sport, do you experience sleep disturbance?
   a. Yes
   b. No

12. When you are travelling for your sport, do you experience daytime dysfunction (feeling generally unwell or having poor performance)?
   a. Yes
   b. No

13. Are you typically a loud snorer?
    a. Yes
    b. No

14. Have you been told that you choke, gasp, or stop breathing for periods of time during sleep?
    a. Yes
    b. No

15. On average, how many caffeinated products (caffeine pills, coffee, tea, soda, energy drinks) do you have per day? For coffee and tea, one drink = 6-8oz/177-237ml; for caffeinated soda, one drink = 1 can (12oz/355ml)?
    a. Less than 1 per day
    b. 1-2 per day
    c. 3 per day
    d. 4 per day
    e. 5 or more per day

16. Over the recent past, how often do you use an electronic device (example: cell phone, computer, tablet, T.V. etc.) within 1 hour of going to bed?
    a. Not at all
    b. 1-3 times per week
    c. 4-6 times per week
    d. Every day
