# Supplementary material for: The Clinical Validation of the Athlete Sleep Screening Questionnaire: an Instrument to Identify Athletes that Need Further Sleep Assessment
Source: Sports Med Open. 2018 Jun 4;4:23. doi: 10.1186/s40798-018-0140-5 (PMC5986689; doi:10.1186/s40798-018-0140-5)
Supplement: Supplementary file 2 — ASSQ sleep difficulty score (SDS) scoring key. (DOCX 22 kb) [file 40798_2018_140_MOESM2_ESM.docx]

Additional file 2

**ASSQ SLEEP DIFFICULTY SCORE (SDS) SCORING KEY**

Add the scores from ASSQ items 1, 3, 4, 5, and 6 to get the SDS. The score corresponds to the following clinical sleep problem category:

**None**: 0-4; **Mild**: 5-7; **Moderate**: 8-10; **Severe**: 11-17

1. During the recent past, how many hours of actual sleep did you get at night? (This may be different than the number of hours you spent in bed.)

a. 5 to 6 hours (4)

b. 6 to 7 hours (3)

c. 7 to 8 hours (2)

d. 8 to 9 hours (1)

e. more than 9 hours (0)

1. How satisfied/dissatisfied are you with the quality of your sleep?

a. very satisfied (0)

b. somewhat satisfied (1)

c. neither satisfied nor dissatisfied (2)

d. somewhat dissatisfied (3)

e. very dissatisfied (4)

1. During the recent past, how long has it usually taken you to fall asleep each night?

a. 15 minutes or less (0)

b. 16 – 30 minutes (1)

c. 31 – 60 minutes (2)

d. longer than 60 minutes (3)

1. How often do you have trouble staying asleep?

a. none (0)

b. once or twice per week (1)

c. three or four times per week (2)

d. five to seven days per week (3)

1. During the recent past, how often have you taken medicine to help you sleep (prescribed or over-the-counter)?

a. none (0)

b. once or twice per week (1)

c. three or four times per week (2)

d. five to seven times per week (3)

**INTERVENTION RECOMMENDATIONS**

The following can be used as a guide for intervention recommendations.

A = General sleep education information sheet specific to athletes + Tailored recommendations depending on the sleep insufficiencies or specific sleep issues

B = Monitoring and follow-up from support team

C = Follow-up and assessment from the sport physician

D = Assessment and recommendations from sleep medicine physician or qualified sleep professional

E = Diagnostic testing for sleep disorder and treatment

**FOR CLINICAL SLEEP PROBLEM CATEGORIES**

**None (SDS of 0-4) –** Recommendation: A

**Mild (SDS of 5-7) –** Recommendations: A + B

**Moderate (SDS of 8-10) –** Recommendations: A + B + C + D + E (if indicated)

**Severe (SDS of 11-17) –** Recommendations: A + B + C + D + E

**MODIFIERS (Sleep Disordered Breathing, Travel, Chronotype)**

The modifiers are not included in the SDS because they occur less frequently and are not always applicable to certain athlete groups (e.g. travel). The modifiers are important to provide specific education and intervention recommendations.

**Sleep Disordered Breathing –** If the athlete answers yes to item 13 (loud snoring) or item 14 (apneas) they should be further evaluated.

Recommendations: D + E (if indicated).

**Sleep and Performance Issues During Travel –** If the athlete answers yes to item 11 (sleep disturbance), education on travel management and jet lag may be necessary.

Recommendations: A (specific to travel).

If the athlete answers yes to item 12 (performance issues), the problem is more serious and could require additional interventions above and beyond education.

Recommendations: A + B + C (if indicated) + D (if indicated).

**Chronotype (Eveningness) –** Those who are evening types are more at-risk for sleep disturbances. Add the scores from items 7-10 to get the Chronotype Score for eveningness. Totals ≤ 4 indicate the athlete is an evening type.

Recommendations: D + E (if indicated).

7. Considering only your own “feeling best” rhythm, at what time would you get up if you were entirely free to plan your day?

a. 5:00 am – 6:30 am (4)

b. 6:30 am – 7:45 am (3)

c. 7:45 am – 9:45 am (2)

d. 9:45 am – 11:00 am (1)

e. 11:00 am – 12:00 pm (noon) (0)

8. How alert do you feel during the first half-hour after having awakened?

a. not at all alert (0)

b. slightly alert (1)

c. fairly alert (2)

d. very alert (3)

9. Do you consider yourself to be a morning type person or an evening type person?

a. definitely a morning type (3)

b. more a morning type than an evening type (2)

c. more an evening type than a morning type (1)

d. definitely an evening type (0)

10. Considering your own “feeling best” rhythm, at what time would you go to bed if you were entirely free to plan your evening?

a. 8:00 pm – 9:00 pm (4)

b. 9:00 pm – 10:15 pm (3)

c. 10:15 pm – 12:30 am (2)

d. 12:30 am – 1:45 am (1)

e. 1:45 am – 3:00 am (0)

**OTHER ITEMS (Items 2, 15, 16)**

These items are not included in the SDS or the modifiers but are important to inform sleep optimization strategies, such as strategies to increase napping frequency, reduce caffeine intake, and reduce electronic device use before bedtime.
